# Investigating the Immunogenic Potential of Variations in Host Cell Protein Levels in Clinical-Grade AAV8 Products

**DOI:** 10.1167/iovs.66.6.38

**Published:** 2025-06-11

**Authors:** Immanuel P. Seitz, Eduardo Rodríguez-Bocanegra, Kirsten Bucher, Felix F. Reichel, Stylianos Michalakis, Dimitri Romanovsky, Martin Biel, Bernd Wissinger, Karl-Ulrich Bartz-Schmidt, Tobias Peters, Manuel D. Fischer, on behalf of the RD-CURE Consortium

**Affiliations:** 1 Centre for Ophthalmology, University Hospital Tübingen, Tübingen, Germany; 2 Oxford Eye Hospital, Oxford University Hospitals NHS Foundation Trust, Oxford, United Kingdom; 3 Nuffield Department of Clinical Neurosciences, University of Oxford, Oxford, United Kingdom; 4 Department of Ophthalmology, University Hospital, LMU Munich, Munich, Germany; 5 Department of Pharmacy, Center for Drug Research, Ludwig-Maximilians-University of Munich, Munich, Germany

**Keywords:** AAV, atrophy, inflammation, gene therapy, nonhuman primates

## Abstract

**Purpose:**

Adeno-associated virus (AAV) formulations for gene therapy contain manufacturing-associated impurities such as residual host cell protein (HCP). The aim of this study was to investigate whether high levels of HCP in AAV formulations are associated with increased inflammation and reduced ocular tolerability.

**Methods:**

Three lots of clinical-grade AAV8 vector were analyzed for the presence of manufacturing-associated impurities. The HCP component of these impurities was characterized using mass spectrometry. Lots were then compared regarding their capacity to induce a cytokine response in primary human plasmacytoid dendritic cells (pDCs) and THP-1 cells. Furthermore, the results of an ocular safety study in healthy nonhuman primates were analyzed post hoc to investigate the influence of HCP levels on clinical signs of inflammation and chorioretinal atrophy (CRA) development.

**Results:**

Vector lots displayed up to a ∼40-fold variation in HCP levels. Human galactin-3-binding protein was the only major HCP contaminant. Stimulation of human pDCs and THP-1 cells with a high HCP lot did not result in an increased cytokine response. High HCP also did not exacerbate clinical signs of inflammation. However, on retinal imaging, CRA lesions were significantly larger in high HCP-treated eyes (*P* = 0.001–0.048).

**Conclusions:**

HCP impurities were of low complexity, but pronounced variations in their abundance were observed between lots. High HCP levels were not overtly immunogenic in vivo and in vitro. However, despite statistical limitations, they seemed to be associated with increased CRA. Thus, a negative effect of high HCP levels on retinal tolerability could not be ruled out.

The approval of voretigene neparvovec (VN) in 2017 and the confirmation of clinical benefits in the real-world setting illustrate the therapeutic potential of retinal gene therapy (GT). Since then, considerable efforts have been undertaken to expand the roster of approved GTs for ocular disorders.^1^ Particular attention has recently been attracted by the emergence of chorioretinal atrophy (CRA) in patients treated with VN.^2^^–^^4^ While the definite cause of the CRA is still being debated,^5^^,^^6^ it is possible that their occurrence is somehow linked to the utilized viral vector: adeno-associated virus (AAV).^7^^,^^8^ This notion was recently strengthened by findings of AAV-dependent atrophies after subretinal injections in nonhuman primates.^8^ Despite its benign reputation, AAV has been demonstrated repeatedly to induce relevant immune responses.^9^^–^^11^ One potential source of AAV immunogenicity is GT manufacturing. Even a clinical-grade AAV product, which has been manufactured to good manufacturing practice (GMP) guidelines, is not devoid of chemical and biological impurities, which could cause or amplify an immune response to the overall product. “Impurities” in this context are defined as any component of a purified AAV suspension other than the desired product (i.e., transgene enveloped in capsid) and are usually remnants of the production process.^12^ This might include free proteins or DNA from host cells used in AAV production such as human embryonic kidney (HEK) or *Spodoptera frugiperda* (Sf9) cells, culture medium components, reagents used for purification, endotoxins, and residual viral or plasmid DNA. If unrecognized, adverse effects due to impurities rather than the therapeutic agent might unnecessarily call AAV-based GT into question. At the same time, removal of such impurities would present a straightforward approach to reduce adverse reactions to current GT approaches. Previous work of our group has demonstrated that residual-free DNA might be an important driver of immune response to GT, which is potentially ameliorable by additional DNase pretreatment of the AAV suspension.^13^ In addition, nucleic acid contaminant-derived antigens have recently been implicated in AAV-mediated liver injury.^14^

Another less investigated impurity is residual host cell protein (HCP). Depending on the production method used, this protein is derived from either HEK or Sf9 cells. Previous work by Rumachik et al.^15^ analyzed 10 different vector lots by the same manufacturer, as well as 13 more AAV vector lots from eight different institutions (private sector, academic core facilities, government consortia, individual laboratories) and found that HCP impurities universally occur across serotypes, manufacturing platforms, production methods, purification protocols, facilities, and scale. Despite their demonstrated ubiquity, knowledge on the clinical relevance of HCP impurities remains limited. A major challenge in narrowing down their clinical relevance is the wealth of different HCP compositions that can occur in AAV preparations. A recent study by Leibiger et al.^16^ identified 2746 unique HEK293-derived protein species copurified across multiple AAV serotypes. Of these HCPs, 1629 (59%) were serotype-specific, while 1117 (41%) were detected in all serotypes (AAV2, AAV5, AAV8, and AAV9). These include molecular chaperones (e.g., HSPA1B), nucleic acid–binding proteins (e.g., nucleolin, nucleophosmin), metabolic enzymes (e.g., alpha-enolase), and components of ribonucleoprotein complexes, all of which may have implications for immunogenicity or vector potency. The purpose of this study was to characterize HCP impurities in clinical-grade AAV lots determined for use in a clinical trial and to investigate their immunogenic potency in vitro and in vivo. For the in vitro experiments, the difference in cytokine response elicited by AAV lots with low versus high concentrations of HCP was evaluated in two relevant cell lines, primary human plasmocytoid dendritic cells (pDCs) and THP-1 cells, which were previously shown to be immunocompetent either to AAV itself or to common HCP impurities.

pDCs are a key antigen-presenting cell in the immune response to viral infections and AAV-based gene therapy.^17^ Upon recognition of double-stranded DNA via toll-like receptor 9 (TLR9), this unique subset of dendritic cells can secrete high levels of type I interferon (IFN), as well as other proinflammatory cyto- and chemokines. The use of pDCs as an AAV-sensing, immunocompetent in vitro cell model was first described by Zhu et al.^18^ Previous work of our group has used the pDC model to investigate TLR9-mediated immunogenicity of various AAV preps.^13^ The THP-1 acute monocytic leukemia cell line has been used to study immune responses due to its ability to detect foreign pathogens, such as bacteria and viruses, via pattern recognition receptors (PRRs) and produce proinflammatory cytokines and chemokines.^19^ THP-1 cells are minimally sensitive to PRR ligands when undifferentiated. However, after differentiation with phorbol 12-myristate 13-acetate (PMA), THP-1 cells acquire a macrophage-like phenotype. These differentiated THP-1 cells can sense some of the main HCP impurities found in AAV lots, such as human galactin-3-binding protein (G3BP),^20^ via toll-like receptor 4 (TLR4). The in vitro experiments were flanked by a post hoc analysis of in vivo ocular safety and toxicity data generated in nonhuman primates (NHPs) with regard to a potential influence of HCP concentration on clinical signs of inflammation and CRA development.

## Methods

### Vector Lots

The experiments in this study used three clinical-grade AAV8 vector lots (Table 1). One of the lots contained a low concentration of HCP and was designated L1. Two lots contained higher concentrations of HCP and were designated H1 and H2. All three lots contained the same AAV8 construct and were manufactured according to current good manufacturing practice (cGMP) requirements at the same facility (Plateforme Atlantic Bio GMP, Nantes, France). The manufacturing process was based on a transient double-transfection protocol of a fully characterized HEK293 Master Cell Bank (MCB). Both plasmids were produced from two high-quality characterized *Escherichia* *coli* MCBs (DH10B strain). Following expansion, HEK293 cells were seeded in CellStacks (Corning, Corning, NY, USA) 10 chambers for the double-transfection step. The transfected cells and supernatant were then harvested in a BioProcess Container (Thermo Fischer Scientific, Waltham, MA, USA), and the lysate was clarified by low-speed centrifugation. The cell pellet was discarded, and the supernatant was polyethylene glycol (PEG)-precipitated overnight at 2–8°C and then stored frozen at ≤−70°C. After thawing, the product was treated with Benzonase (Merck Millipore (now MilliporeSigma), Burlington, MA, USA) to digest nucleic acids. It was then purified by two rounds of cesium chloride gradient ultracentrifugation, followed by a tangential flow filtration step for diafiltration and concentration. After formulation, the resulting vector lots were stored at ≤−70°C. In-process samples were collected during the entire manufacturing process for potency, identity, purity, and safety assays.

**Table 1.** Comparative Analysis of AAV8 Vector Lot Impurities

Lot Designation	Low HCP (L1)	High HCP 1 (H1)	High HCP 2 (H2)
Residual HCP-HEK293 protein ELISA (ng/mL)	36.9	1433.7	582.0
Lot used for	In vitro + in vivo	In vitro	In vivo
Other			
Full/empty capsid ratio (%)	99.5	98.5	96.0
Sterility	Sterile	Sterile	Sterile
Endotoxin (EU/mL)	<0.0150	<0.0150	<0.0150
Titration vector genomes qPCR (vg/mL)	5.9 × 10E12	6.0 × 10E12	6.1 × 10E12
Protein purity (SDS-PAGE/Coomassie)	87.8%	88.4%	84.0%
Residual cell DNA albumin qPCR (ng/mL)	<LOQ	<LOQ	<LOQ
Total protein (Bradford) (µg/mL)	11.1	15.7	14.8
Residual BSA protein ELISA (ng/mL)	<LOQ	<LOQ	<LOQ
Residual Benzonase ELISA (ng/mL)	<LOQ	<LOQ	<LOQ
Residual cesium chloride (ICP/MS) (µg/mL)	29.2	45.0	88.4
Residual PEG 8000 (HPLC/CAD) (µg/mL)	<LOQ	<LOQ	<LOQ
Residual E1A cell DNA E1A qPCR (copy/mL)	1.7 × 10E4	4.5 × 10E4	4.5 × 10E4
Residual plasmid DNA Kana-R qPCR (copy/mL)	1.2 × 10E10	9.9 × 10E9	1.1 × 10E10
Osmolality EP 2.2.35 (mOsm/Kg)	311	310	317
pH	7	7	7.1

All AAV8 vector lots used in this study underwent rigorous quality control in accordance with GMP guidelines. The principal difference between lots was found in their residual HCP content (i.e., 39- and 16-fold variance). No other parameter displayed relevant variation between lots. Slight discrepancies were noted in residual cesium chloride levels and residual E1A cell DNA (<3-fold), albeit at a very low absolute level. CAD, charged aerosol detector; HPLC, high performance liquid chromatography; ICP, inductively coupled plasma mass spectrometry; LOQ, limit of quantification; qPCR, quantitative PCR.

### Host Cell Protein Quantification

Lot characterization was performed to GLP standards by the Gene Therapy Laboratory IJMR1089, at the University of Nantes (France), and Protim, a proteomic platform of the French Institute de recherche en santé, environnement et travail (Irset), located in Rennes (France). Residual HCP levels in the three vector lots were determined with a commercially available immunoassay from Cygnus Technologies (Leland, NC, USA) (#F650). This HEK293 assay is a two-site immunoenzymetric assay. Samples containing HEK293 HCP were reacted simultaneously with a horseradish peroxidase–labeled anti-HEK antibody (goat polyclonal) in microtiter strips coated with an affinity-purified capture anti-HEK antibody. The immunological reaction resulted in the formation of a sandwich complex of solid-phase antibody reactants. The substrate 3,3′,5,5′-tetramethylbenzidine was then reacted. The amount of hydrolyzed substrate was read on a microtiter plate reader at 450/630 nm and is directly proportional to the concentration of HEK293 HCP present in the vector lots. Lot samples were applied to the ELISA kit. To verify that no inhibitor interferes with the assay, lot samples spiked with a known amount of HEK293 HCP were used as additional controls.

### Host Cell Protein Identity

The protein content of all AAV8 vector lots was analyzed via nano–liquid chromatography coupled to tandem mass spectrometry (nanoLC-MS/MS). The aim of this analysis was to compare the protein content between the three lots and to identify the composition of HEK293 cell residual proteins. One vial per lot was thawed on ice. Total proteins were precipitated by the addition of four volumes of ice-cold acetone, incubated overnight at −20°C, and pelleted by centrifugation at 16,000 × *g* for 15 minutes. Protein pellets were dissolved in 20 µL 1× NuPAGE LDS sample buffer. The 20-µL protein samples were loaded on a 12% SDS-polyacrylamide gel and submitted to a short electrophoresis (3 minutes at 200 V). Proteins were then stained with Coomassie blue, and protein-containing gel slices were retrieved from the gel. Gel slices were submitted to different chemical pretreatments, including reduction and alkylation of disulfide bonds, and then to in-gel trypsin digestion. Proteolytic peptides were finally extracted from the gel slices, and final sample volumes were adjusted to 30 µL. For each of the proteolytic peptide samples, 10 µL was submitted to nanoflow high-performance liquid chromatography (nanoLC) coupled to tandem mass spectrometry (MS/MS). LC-MS/MS data were analyzed using Mascot Distiller software and identified using the Mascot search engine. For protein identification, peptides were queried against two databases: the UniprotKB human proteins database and a vector-specific database of viral (adenovirus and AAV) proteins expressed during AAV8 production in HEK293 cells. Results are expressed using the exponentially modified Protein Abundance Index, a quantitative value, which is indicative of the relative amount of protein in complex samples.^21^

### Isolation and Purification of Primary Human pDCs

Human pDCs were obtained from buffy coats of healthy young donors from the Centre for Clinical Transfusion Medicine (University of Tübingen, Tübingen) after approval by the local ethics board of the University Hospital Tübingen. Peripheral blood mononuclear cells (PBMCs) were isolated using Ficoll density gradient centrifugation (Ficoll-Paque; GE Healthcare, Uppsala, Sweden) for 35 minutes at 400 × *g*. pDCs were purified from PBMCs by magnetic activated cell sorting by negative selection using biotin-conjugated antibodies and antibiotin microbeads (Miltenyi Biotec, Bergisch Gladbach, Germany). Flow cytometry was performed to assess the pDC purity of the protocol. Cell samples of 100 µL were stained using PE Mouse Anti-Human CD123 (dilution 1:80) and BV421 Mouse Anti-Human BDCA-2 CD303 (dilution 1:40) antibodies (RRID: AB_395457, AB_2744264; BD Biosciences, Franklin Lakes, NJ, USA). Measurement was performed on a FACSCanto II (BD Biosciences, Franklin Lakes, NJ, USA), and data were evaluated with FlowJo software.

### Stimulation of Primary pDCs With Low and High HCP AAV8 Vector Lots

pDCs were seeded at 1.25 × 10E4 cells/well on 384-well plates (Corning, Corning, NY, USA) in medium containing RPMI 1640 (Sigma-Aldrich, St. Louis, MO, USA) supplemented with 10% HS (heat-inactivated foetal bovine serum, Thermo Fischer Scientific, Waltham, MA, USA), 1% GlutaMAX (Thermo Fischer Scientific, Waltham, MA, USA), 1% MEM Non-Essential Amino Acids (NEAA; Gibco, Thermo Fischer Scientific, Waltham, MA, USA), 1% Sodium Pyruvate (Gibco), and 1% penicillin-streptomycin (Thermo Fischer Scientific, Waltham, MA, USA). After seeding, cells were stimulated with the TLR-9 agonist ODN2216 (Biomers.net, Ulm, Germany) or two different lots of AAV8 vector at a multiplicity of infection (MOI) of 1 × 10E6 vg, containing either low (L1) or high (H1) levels of HCP. Medium-only pDCs served as a baseline. After 18 hours of incubation at 5% CO_2_, 37°C, supernatants were collected and kept at −80°C until being used. The MOI of 1 × 10E6 was chosen after previous works in the pDC model had shown cytokine reactivity using it^13^ and because it reasonably approximates a high-dose treatment in subretinal gene therapy. Low (L1) and high (H1) HCP lots were compared to medium-only controls. To characterize the TLR9-dependent cytokine response of primary pDCs, they were stimulated with TLR9 agonist ODN2216 (Supplementary Fig. S1). Supernatants were analyzed using a Bio-Plex Pro Human Cytokine Panel (cat. L500JMN744; Bio-Rad, Hercules, CA, USA) with a total of 19 targets: IP-10, MIP-1β, TNF-α, IFN-α, IL-1β, IL-2, IL-4, IL-5, IL-6, IL-7, IL-8, IL-10, IL-12, IL-13, IL-17, G-CSF, GM-CSF, IFN-γ, and MCP-1. These magnetic bead–based Multiplex assays were performed on a Luminex 200 system (Bio-Rad) in accordance with the manufacturer's instructions. Standards were analyzed in duplicates and samples in triplicates. Analysis of the data was performed using Bio-Plex Manager (Bio-Rad). Secretion of inflammatory cytokines in response to AAV8 vector lots was measured using cytokine-specific sandwich-ELISA kits (cat. DY266-05, DY271-05, DY208-05 [R&D Systems, MN, USA] and cat. 555212 [BD Biosciences]) according to the manufacturer's instructions. Absorbance was quantified using a M200 NanoQuant spectrophotometer (Tecan, Männedorf, Switzerland) at 450 nm (570 nm correction wavelength). Results were calculated using GraphPad Prism (GraphPad Software, La Jolla, CA, USA) by applying a 4PL regression model.

### Differentiation of THP-1 Cells and Stimulation With a High HCP AAV8 Vector Lot

Cells from a THP-1 monocyte cell line were purchased from ATCC (Manassas, VA, USA). They were cultured in THP-1 medium consisting of RPMI 1640 (Sigma-Aldrich, St. Louis, MO, USA) supplemented with 10% heat-inactivated fetal bovine serum (Gibco, Thermo Fischer Scientific, Waltham, MA, USA), 1% GlutaMAX (Gibco), and 1% penicillin/streptomycin (Gibco). Cells were maintained in the incubator at 5% CO_2_, 37°C. Before stimulation experiments, cells were seeded at a density of 5 × 10E4 cells/well in 96-well plates (Corning) and differentiated with PMA (Sigma-Aldrich) at a concentration of 100 nM in THP-1 medium. Differentiation was evident after 48 hours when cells became adherent, proliferation stopped, and they phenotypically resembled macrophages. Following differentiation, fresh THP-1 medium without PMA was added, and cells were kept in the incubator for another 24 hours. For stimulation experiments, differentiated THP-1 cells were incubated with either an AAV vector lot at an MOI of 1 × 10E6 or the TLR4-ligand lipopolysaccharide (LPS; Invivogen, San Diego, CA, USA) at 10 ng/mL. Cells were incubated for 24 hours, followed by supernatant collection. Both AAV and LPS were compared to THP-1 cells, which received no treatment (i.e., medium only). Cytokine and chemokine release was quantified as described previously for pDCs.

### In Vivo Data on the Influence of HCP Levels in NHPs

To cross-validate our in vitro findings, we reanalyzed data from a two-part, GLP-compliant (good laboratory procedures), IND-enabling (IND: investigational new drug) study for a novel gene therapy for *PDE6A-*related retinitis pigmentosa in NHPs with regard to a potential influence of HCP levels on adverse outcomes in vivo. The methodology for the abovementioned NHP study, the imaging procedures, and the grading of inflammatory reaction have been published previously.^8^^,^^22^ In brief, animals were treated subretinally with either a low (1 × 10E11) or high (1 × 10E12) dose of AAV and observed for 13 weeks. Both low- and high-dose eyes received the same subretinal volume, with the low dose being a dilution of the high dose. This article reports a retrospective analysis on the influence of low versus high HCP lots on clinical findings in these NHPs. This post hoc analysis was enabled by the fact that, in addition to the different AAV dosages, animals were also treated with two vector lots that contained different levels of HCP. One cohort was treated with lot L1 (37 ng/mL) and the other with H2 (582 ng/mL). The evaluated endpoints were lesion size in fundus autofluorescence (FAF) (i.e., atrophy of the retinal pigment epithelium), fluorescein angiography (FA) (window defects), and indocyanine green angiography (ICGA) (choroidal hypoperfusion) [mm^2^] (Supplementary Fig. S2), as well as signs of inflammation on slit-lamp examination. Anterior chamber cells (ACCs) and vitreous cells (VCs) were graded using the semi-quantitative Standardization of Uveitis Nomenclature working group scale. Of note, other signs of inflammation, such as conjunctival hyperemia and chemosis, were not reported, as these signs were not recorded in any animal after day 4 postsurgery (i.e., likely procedure related). The NHP studies were conducted at the Labcorp Early Development Services GmbH facility in Münster, Germany, in adherence to Directive 2010/63/EU for the protection of animals used for scientific purposes and the 2007/526/EC Commission Recommendation (Appendix A of Convention ETS 123). The studies were in compliance with the German Animal Welfare Act, as well as the ARVO Statement for the Use of Animals in Ophthalmic and Vision Research, and approved by the local Institutional Animal Care and Use Committee (LANUV).

### Statistical Analysis

Statistical analysis was performed using GraphPad Prism 10.0.0 for Mac (GraphPad Software, La Jolla, CA, USA) and JMP 18.0 (SAS Institute, Cary, NC, USA). Unless specified otherwise, continuous variables are expressed as mean ± SD. One-way ANOVA was performed in GraphPad Prism to investigate differences in cytokine release between different treatment groups in vitro. Due to the small sample size in the NHP experiments, a statistician was consulted to select and validate appropriate statistical tests. Various tests appropriate for continuous variables (e.g., linear mixed-effects models) had to be discarded due to the small sample size and violations of key test assumptions. Ultimately, differences between NHP treatment groups were investigated in JMP18 using the nonparametric Wilcoxon rank-sum test for continuous variables (e.g., CRA lesion size) and Pearson's χ^2^ test and likelihood ratio for ordinal variables (e.g., clinical inflammation scores).

## Results

### Comparative Analysis of AAV8 Vector Lot Impurities

Three AAV8 vector lots were used to probe in vitro and in vivo the immune response to elevated levels of HCPs. Table 1 illustrates the analytical results for all three involved AAV8 lots, including HCP concentration. The AAV lot with the lowest concentration of HCP was designated L1 (L for low HCP). Compared to L1, HCP levels in the other two lots were elevated ∼40-fold and ∼15-fold, respectively. These lots were therefore designated H1 and H2 (H for high HCP). Apart from the different HCP levels, there was slight variation (2.5- to 3-fold) in residual cesium chloride and helper virus DNA left over from the AAV production (E1A DNA), albeit at a very low overall level. All further impurities displayed no relevant variation (<2-fold) between lots. L1 was the least impure lot by all parameters.

### Characterization of HCPs

NanoLC tandem mass spectrometry was performed to elucidate protein identity (Table 2). The HCP impurities were found to be of low complexity. Only two HEK-derived protein species were detected in relevant quantities: (1) human G3BP, which was the major contaminant and occurred in all tested lots, and (2) polyubiquitin B (UBB), which was only found in lot H1 in low concentrations. As expected, the most abundant protein in all three lots was the AAV serotype 8 capsid protein VP1. It was not possible to specifically detect the small VP proteins (i.e., VP2 and VP3) as their sequences overlapped with VP1. No other viral protein was detected. In addition, the samples contained multiple keratins of environmental origin.

**Table 2.** Protein Characterization by Lot

	Protein Abundance [emPAI]		
Protein Description	L1	H1	H2
Viral protein			
AAV8 VP1	8.65	8.18	10.86
HEK-cell protein			
Human G3BP	0.67	1.15	1.02
UBB	0	0.57	0
Protein of environmental origin			
Keratin, type II cytoskeletal 1 (KRT1)	1.96	1.02	1.02
Keratin, type I cytoskeletal (KRT10)	1.53	1.19	0.43
Keratin, type II cytoskeletal 2 epidermal (KRT2)	1.16	0.90	0.47
Keratin, type I cytoskeletal 9 (KRT9)	0.72	0.61	0
Keratin, type II cytoskeletal 6A (KRT6A)	0	1.01	0.15
Keratin, type I cytoskeletal 16 (KRT16)	0	0.92	0
Keratin, type I cytoskeletal 14 (KRT14)	0	0	0.38

All AAV8 lots (low HCP: L1, high HCP 1: H1, and high HCP 2: H2) were investigated using nanoLC-MS/MS. Abundance of individual proteins is quantified using the exponentially modified Protein Abundance Index (emPAI). By category: viral protein, HEK cell protein, environmental protein (i.e., human skin and hair contamination). Overall, all lots were highly purified and of low complexity, with only 10 different proteins detected in total. In line with this, AAV8 VP1 was by far the most abundant protein overall. The most abundant host cell protein (i.e., originating in HEK cells) was human galectin-3-binding protein.

### In Vitro Stimulation of pDCs and PMA-Differentiated THP-1 Cells With a High HCP AAV8 Lot Does Not Elicit an Increased Cytokine Response

**Figure 1.**
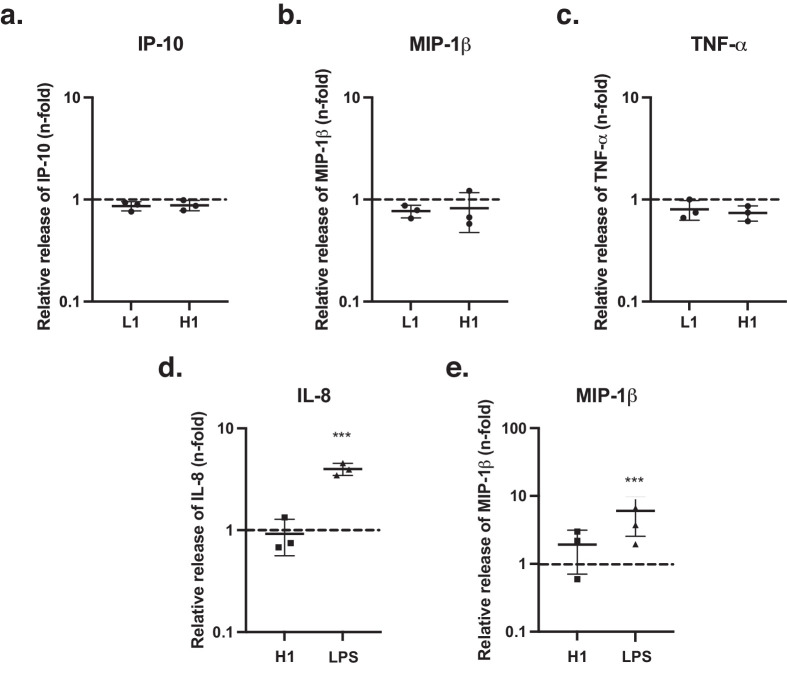
Evaluation of immune response to AAV8 lots containing different HCP levels in pDCs (**a–c**) and PMA-differentiated THP-1 cells (**d**, **e**). (**a**–**c**) Fold increase of cytokine release of IP-10, MIP-1β, and TNF-α after stimulation of pDCs with AAV8-RK-hPDE6A vector lots (MOI: 1:1 × 10^6^ vg) carrying different HCP contents (L1: 36.9 ng/mL, H1: 1433.7 ng/mL), relative to medium-only control. (**d**, **e**) Fold increase of cytokine release of IL-8 and MIP-1β over baseline for the TLR-4 agonist LPS and lot H1 relative to a medium-only control. *Error bars* indicate the standard deviations between replicate assays. Statistical significance was determined using an unpaired Student’s *t*-test. *P ≤ 0.05.

To confirm that the primary pDCs isolated from healthy donors were TLR9 competent, they were seeded and stimulated with the TLR9 ligand ODN2216. ODN2216 induced reactive cell proliferation in the stimulated pDCs. In addition, a significant release of proinflammatory cytokines (IP-10, MIP-1β, TNF-α) and type I IFN (IFN-α) was detected, compared to vehicle control (Supplementary Fig. S1). No significant release was found in the other 15 measured cytokines. In contrast, incubation of pDCs with the low HCP (L1) and high HCP (H1) vector lots did not result in an increased secretion of IP-10, MIP-1β, and TNF-α compared to a medium-only control (Figs. 1a–c). In the THP-1 cell line, the lot with the highest host cell protein concentration (H1) was compared to a medium-only control and the known TLR4-agonist LPS. While LPS induced a robust cytokine response compared to a medium-only control (Figs. 1d, 1e), no significant changes in the release of inflammatory cyto- and chemokines were detected upon stimulation with the high HCP lot H1.

### Influence of Elevated HCP Levels on Adverse Reactions In Vivo

**Figure 2.**
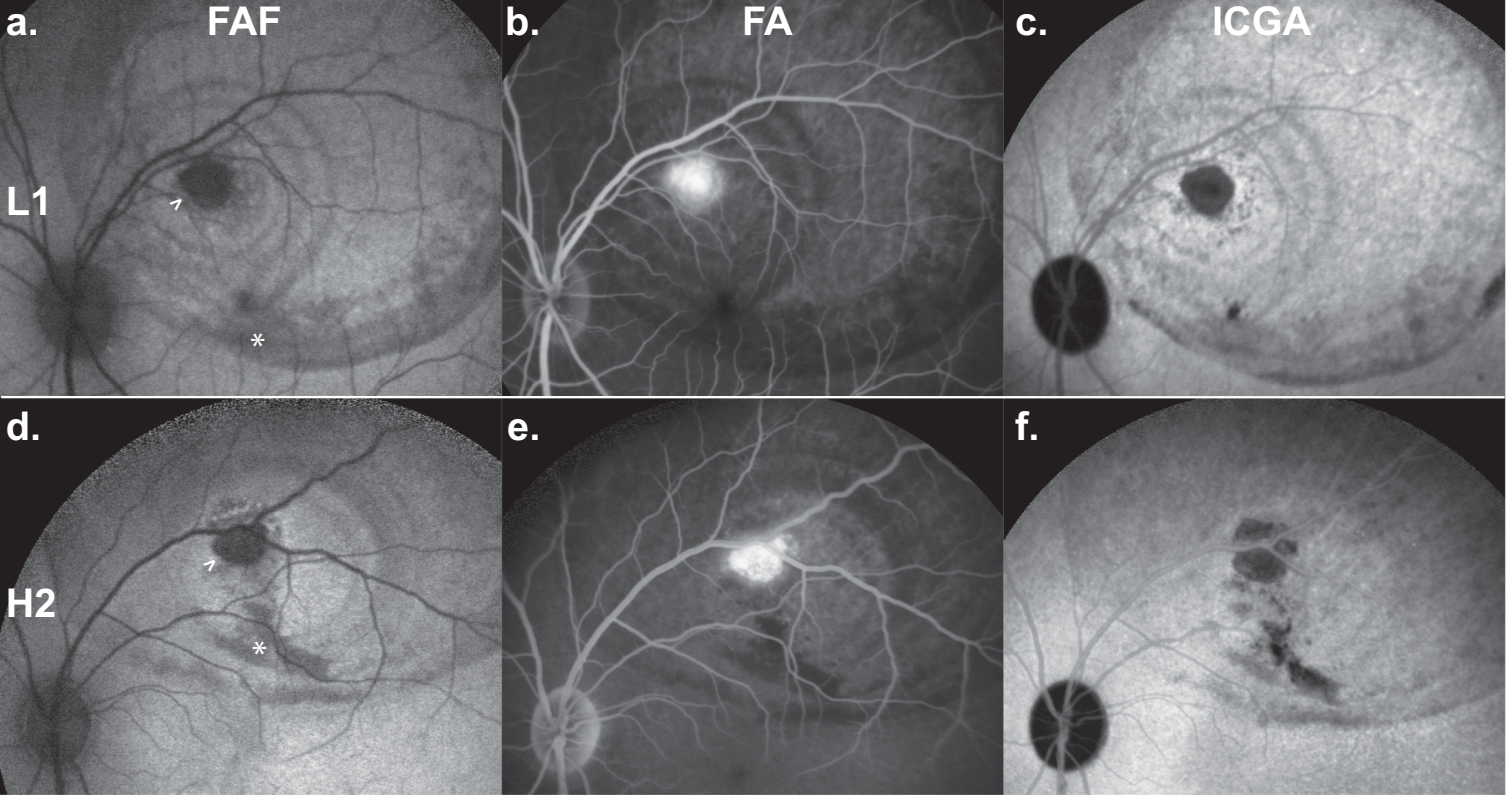
Multimodal imaging of CRA at the injection site. Representative lesion examples for low (**a**–**c**) versus high (**d**–**f**) HCP levels. CRA at the injection site (>) was recorded using (**a**, **d**) FAF, (**b**, **e**) FA, and (**c**, **f**) ICGA. Findings related to the subretinal injection included mechanical displacement of pigment (*). Qualitatively, there was no obvious difference between CRA in eyes treated with a low HCP (L1) versus a high HCP (H2) lot.

**Figure 3.**
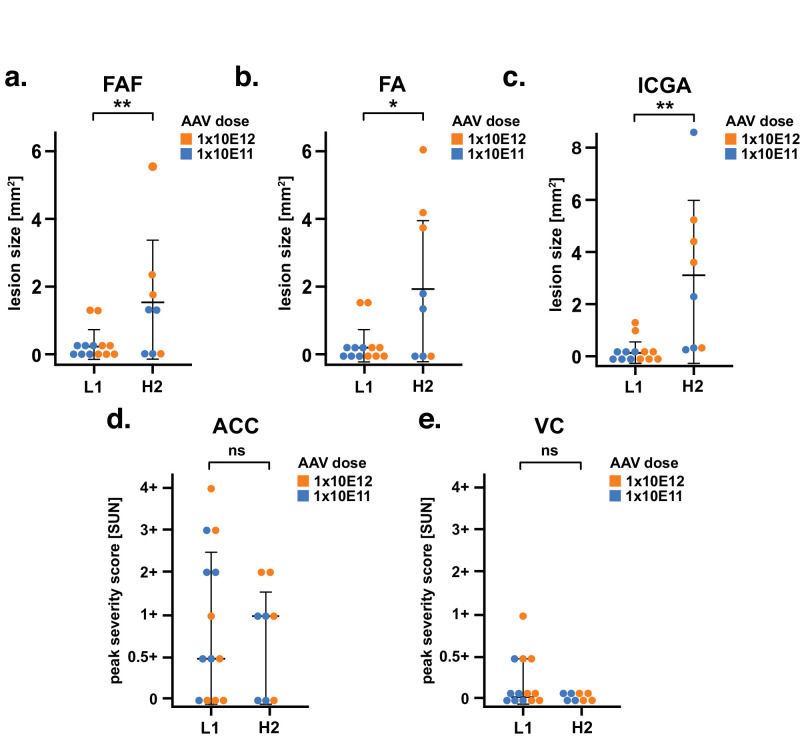
Clinical findings after subretinal injection of AAV8 lots containing different HCP levels in nonhuman primates, color-coded by AAV dose level. (**a–c**) Retinal imaging and (**d**, **e**) slit-lamp exam. (**a**–**c**) Inflammatory and atrophic changes were detected at the injection site 13 weeks after subretinal delivery of AAV in a subset of treated animals. The injection site lesion size was quantified using (**a**) FAF, (**b**) FA, and (**c**) ICGA. There was a statistically significant difference between L1- and H2-treated eyes. This was supported by a trend toward larger lesion size in animals treated with lot H2. *Error bars*: mean ± SD. *P ≤ 0.05, **P ≤ 0.01. (**d**, **e**) Peak clinical inflammatory activity after treatment was determined by quantifying the number of cells in the ACC and VC using the Standard of Uveitis Nomenclature (SUN) grading scheme. There was no difference in overt inflammation between L1- and H2-treated eyes. *Error bars*: median ± interquartile range. ns, not significant. Statistical significance was determined using the Wilcoxon rank-sum test. L1: vector lot with low HCP (37 ng/mL). H2: vector lot with high HCP (582 ng/mL).

On slit-lamp examination, treatment with the high HCP lot H2 was not associated with increased peak ACC and VC scores compared to treatment with the low HCP lot L1 (Figs. 3d, 3e), irrespective of AAV dosage. Multimodal retinal imaging revealed no qualitative difference in CRA lesions between eyes (Fig. 2). Regarding lesion size, a pooled analysis of all L1- versus all H2-treated eyes irrespective of AAV dose was performed (Figs. 3a–c). The mean ± SD CRA lesion size at week 13 after treatment on FAF was 0.2 ± 0.5 mm^2^ for L1-treated vs. 1.5 ± 1.8 mm^2^ for H2-treated eyes (**P = 0.007). For FA, it was 0.3 ± 0.6 mm^2^ for L1-treated vs. 2.0 ± 2.1 mm^2^ for H2-treated eyes (*P = 0.048). ICGA was 0.2 ± 0.4 mm^2^ for L1-treated vs. 3.2 ± 2.9 mm^2^ for H2-treated eyes (**P = 0.001). In addition, a Bonferroni-corrected subgroup analysis stratified by AAV dose (Supplementary Fig. S2) was performed. Within AAV dose groups, differences between L1- and H2-treated eyes were mostly not statistically significant.

## Discussion

As expected, all three GMP grade AAV8 vector lots analyzed in this study contained some amount of HCP. Using nanoLC-MS/MS, these impurities were found to be of low complexity. As expected, VP1 was by far the most abundant protein component in all three lots. The major HEK-derived contaminant was G3BP, which has been described previously in AAV formulations.^23^^–^^26^ A study by Denard et al.^26^ previously reported that G3BP poorly interacts with AAV8, suggesting that it is copurified with the AAV8 particles but not necessarily adsorbed onto the capsids. G3BP is a secreted glycoprotein found in body fluids that interacts with various proteins, in particular, galactose-specific lectin 3 (galectin-3) and proteins of the extracellular matrix. It can form dimers and oligomers, and these oligomers can form ring-shaped structures of 30 to 40 nm, which can interact with AAV capsids^24^ or copurify with vector particles. G3BP contamination was also found to reduce AAV6 vector potency following systemic administration in mice. A similar interaction of G3BP with AAV serotype 8 capsids has not been reported, but it also cannot be excluded. The other identified HEK293-derived host cell protein, UBB, was detected as a minor contaminant exclusively in lot H1. UBB is a ubiquitously expressed intracellular protein that can be conjugated to various target proteins by cellular ubiquitin ligases. Its major function is to target proteins for degradation by the 26S proteasome. Ubiquitination of AAV capsids following cell entry has been shown for different capsid serotypes, and we can hypothesize that this ubiquitination can also occur within HEK293 cells during vector production. In addition, seven human keratins (KRT1, KRT2, KRT6A, KRT9, KRT10, KRT14, KRT16) were detected in our samples. These cytokeratins of epidermal origin (skin or hair cells) did most probably come from environmental contamination during sample preparation, despite all precautions taken. This type of contamination is a common pitfall in nanoLC-MS/MS experiments due to the extraordinary sensitivity of the method. Indeed, the keratins that are reported to be expressed in HEK293 cells (KRT5, KRT8, and KRT18)^27^^–^^29^ were not detected in any of the lots. Currently, regulatory agencies do not specify exact permissible levels of HCPs in gene therapy products. While the relative differences in HCP content among the vector lots in this study were notable, it is essential to contextualize these absolute values within the broader landscape of AAV vector manufacturing. Previous studies employing HEK293-based production systems and standard purification methods have reported residual HCP levels in the range of approximately 10 to 100 ng/mL per 1 × 10E12 vg.^30^ Given the AAV concentration of 6 × 10E12 vg/mL for lots L1, H1, and H2, this translates to a general expectation of 16 to 160 ng/mL HCP in our final products. Thus, the level observed in L1 (37 ng/mL) falls within this expected range, consistent with a well-purified vector preparation. In contrast, the levels in H2 and H1 (582 ng/mL and 1434 ng/mL, respectively) exceed this range substantially (ca. 7- and 16-fold), indicating a comparatively high HCP burden. The designation of “high” and “low” HCP lots therefore seems valid for both the interlot comparison and the generally expected HCP levels in vector production. Furthermore, the composition of HCP impurities can vary significantly depending on the manufacturing platform, AAV serotype, and analytical methodologies employed. In this study, however, all three vector lots were produced within the same facility, and G3BP was the only major contaminant detected across all lots. As such, caution is warranted when trying to generalize these findings, as they cannot predict the biological effects other HCP impurities might have. Nevertheless, G3BP is a commonly observed HCP contaminant that has been identified in formulations of multiple AAV serotypes, including AAV1, AAV2, AAV5, AAV6, AAV8, and AAV9,^16^^,^^26^^,^^31^ and literature on the biological effects of relevant HCP impurities in the eye is sparse. Thus, these findings may nonetheless hold broader relevance for a wide range of gene therapy products.

In vitro stimulation of primary pDCs with the vector lot H1, which contained the highest measured HCP level of 1434 ng/mL, did not elicit or exacerbate significantly the TLR9-mediated immune response to the AAV8 vector. In addition, there was no evidence that the increased presence of TLR4-ligand G3BP was sufficient to trigger the release of inflammatory cytokines in TLR4-competent PMA-differentiated THP-1 cells. However, there is natural variation in primary cell cultures, such as primary pDCs, and detecting weak stimuli in PMA-differentiated THP-1 cells is a known challenge. Therefore, the absent reaction of these cellular models to high HCP lots does not rule out potential adverse effects. In line with the in vitro results, slit-lamp examination did not reveal a significant difference in overt clinical inflammation. Anterior chamber and vitreous cell scores appeared insensitive to HCP concentration. The same was true for conjunctival hyperemia and chemosis, which were not observed past day 4 after surgery.

In contrast, retinal imaging findings were more equivocal. In a previous analysis, development of CRA at the injection site was described in a subset of treated NHPs,^8^ and the size of these lesions was shown to be dependent on AAV dosage. The presented post hoc analysis was conducted to assess whether atrophic lesion was also associated with HCP levels. This expansion beyond the study's original scope (i.e., dose finding) was enabled by two key factors: first, there was a surprising difference in the HCP contents between vector lots. An original toxicology lot (L1, 37 ng/mL) was followed by a first lot destined for clinical use (H1, 1434 ng/mL). The high HCP content of lot H1 then prompted the production of a second lot destined for clinical use (H2, 582 ng/mL). Furthermore, comparable numbers of eyes were treated with vector lots L1 (6 low-dose AAV, 7 high-dose AAV) and H2 (4 low-dose AAV, 4 high-dose AAV). A pooled analysis by HCP (i.e., including both AAV dose groups) revealed a statistically significant association between a higher HCP level and increased lesion size on retinal imaging. This finding was supported by a descriptive trend toward larger CRA lesions in eyes receiving the high HCP lot, particularly in combination with the high AAV dose (Figs. 3a–c). Within AAV dose groups, a definitive, independent adverse effect of high HCP levels could not be demonstrated: in Bonferroni-corrected subgroup analyses (Supplementary Fig. S2), differences between low and high HCP groups were mostly not statistically significant. Given the limitations of post hoc testing and the small sample size, these results should be interpreted with caution.

In summary, up to 40-fold variations in residual HCP content were found in three otherwise similar lots of GMP-grade AAV8 vector, produced in the same facility. Mass spectrometry revealed that the HCP impurities were of low complexity, with G3BP as the only major contaminant. In vitro stimulation of two cell models, one competent for TLR9 (AAV) and one for TLR4 (G3BP), did not reveal an increased release of cytokines when subjected to the lot with the highest HCP concentration. In line with the in vitro findings, a retrospective analysis of ocular safety data from a GLP-compliant NHP study revealed no increased signs of inflammation on slit-lamp examination. Conversely, retinal imaging analysis, although constrained by statistical limitations, indicated a possible link between elevated HCP levels and greater CRA lesion size. Therefore, an adverse biological effect of HCP impurities could not be excluded with certainty. This highlights the critical importance of high-quality manufacturing in delivering safe and effective gene therapy products.

## Supplementary Material

Supplement 1

## Appendix A. Appendix. The RD-CURE Consortium

Members of the RD-CURE Consortium (in institutional and alphabetical order): Sylvia Bolz, Susanne Kohl, Laura Kühlewein, Regine Mühlfriedel, Jonas Neubauer, Alex Francois Paquet-Durand, Mathias Seeliger, Vithiyanjali Sothilingam, Katharina Stingl, Marius Ueffing, Nicole Weisschuh, Bernd Wissinger, Ahmad Zhour, Ditta Zobor, and Eberhart Zrenner (Centre for Ophthalmology, University of Tuebingen, Germany); Stylianos Michalakis (Department of Ophthalmology, University Hospital, LMU Munich); Martin Biel and Christian Schön (Department of Pharmacy, Center for Drug Research, Ludwig-Maximilians-Universität München, Germany); Nadine Kahle and Barbara Wilhelm (STZ eyetrial at the Centre for Ophthalmology, University of Tübingen, Germany); Steven Tsang (Department of Ophthalmology, Columbia University, New York, USA); and Christian Johannes Glöckner (Deutsches Zentrum für Neurodegenerative Erkrankungen, Tuebingen, Germany).
