# Supplementary material for: Investigating the Immunogenic Potential of Variations in Host Cell Protein Levels in Clinical-Grade AAV8 Products
Source: Invest Ophthalmol Vis Sci. 2025 Jun 11;66(6):38. doi: 10.1167/iovs.66.6.38 (PMC12166503; doi:10.1167/iovs.66.6.38)
Supplement: Supplement 1 [file iovs-66-6-38_s001.pdf]

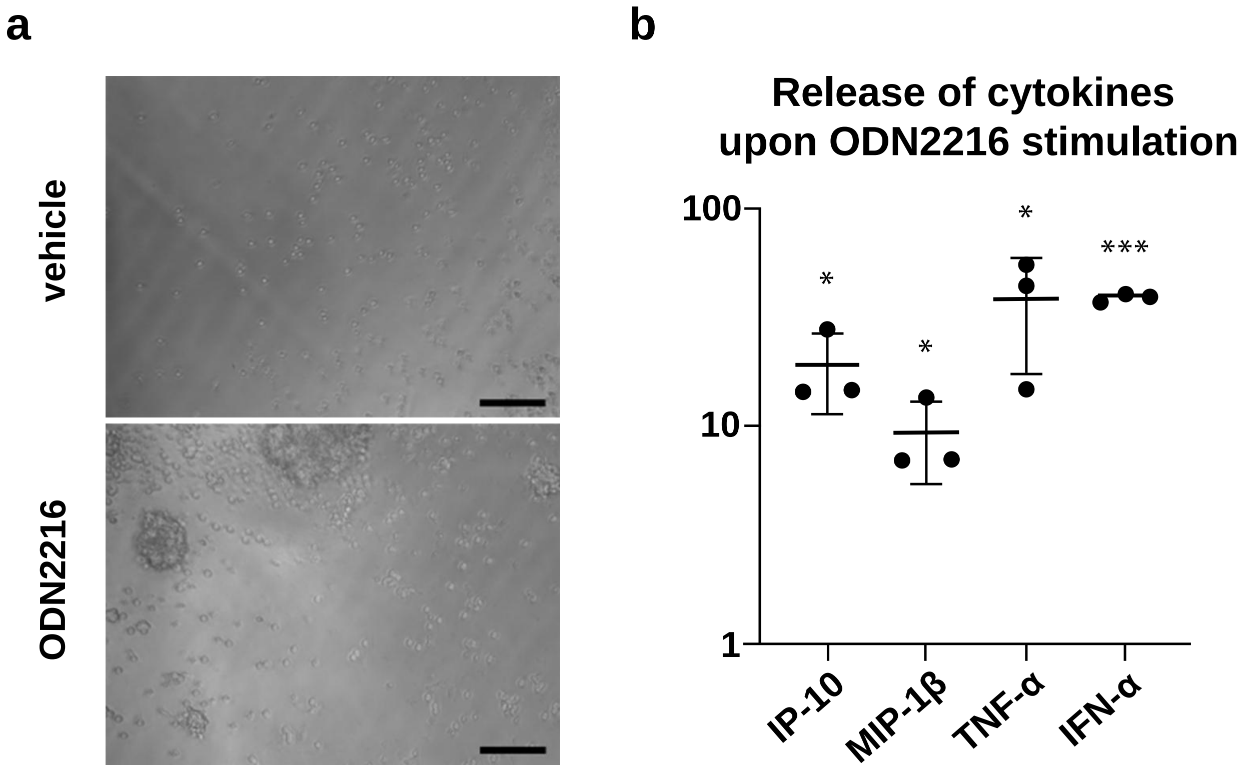

Figure S1: Isolated primary pDCs are immunocompetent. Purified pDCs were stimulated with 0.77 µM ODN2216 for 18 hours. (a) Representative bright field images of pDCs stimulated with vehicle control (*upper image*) or ODN2216 (*lower image*). Scale bar is 100 µm. (b) Fold increase of cytokine release of IP-10, MIP-1β, TNF-α and IFN-α2 by ODN2216-stimulated pDCs. Error bars indicate the standard deviations between replicate assays. Statistical significance was determined using unpaired Student t-test. P-values: ≤0.05: *; ≤0.001: ***.

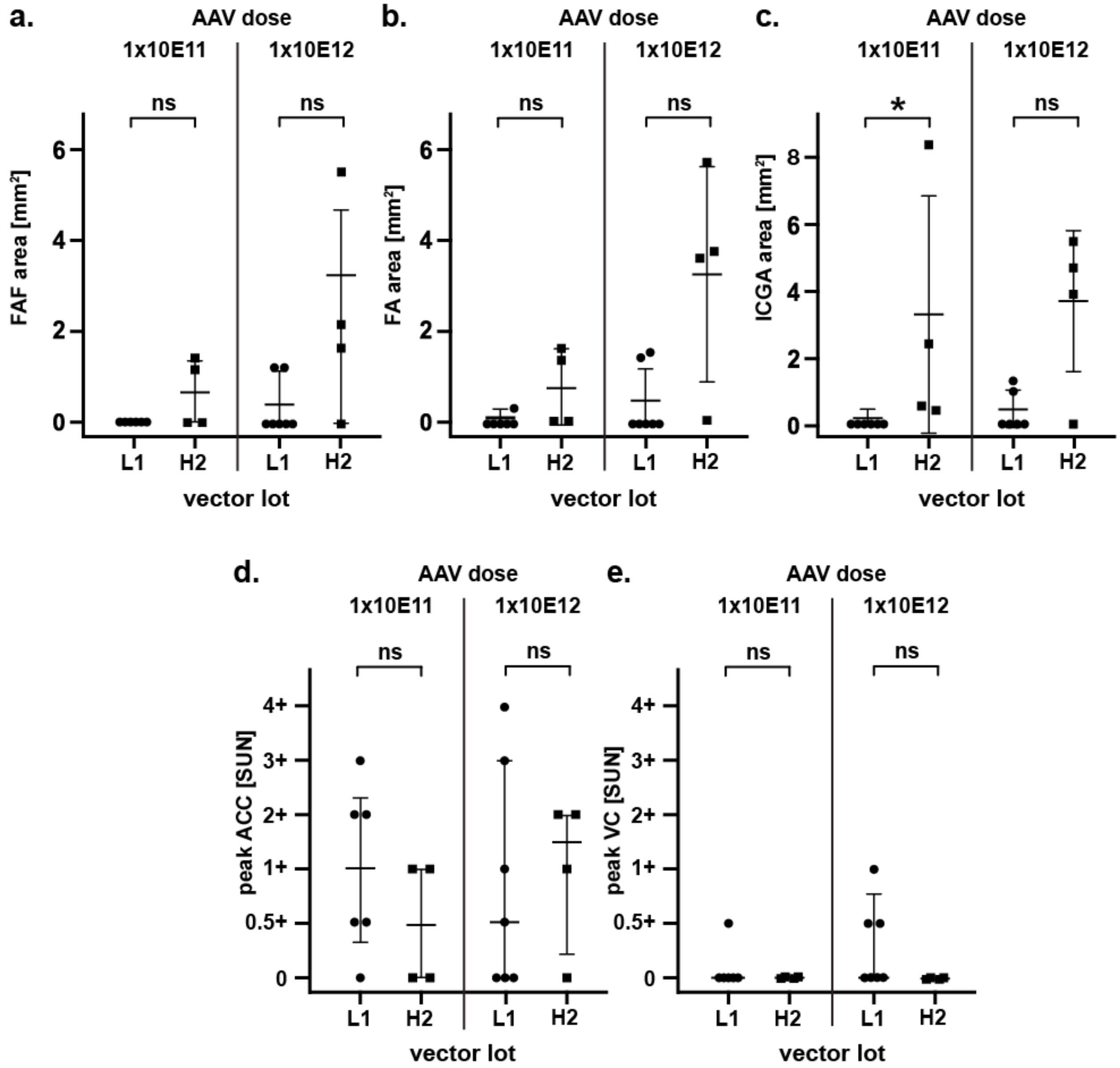

Figure S2: Clinical findings after subretinal injection of AAV8 lots containing different HCP levels in non-human primates, subgroup analyses by AAV dose level. a.-c.: retinal imaging, d.+e: slit lamp exam. a.-c.: Inflammatory and atrophic changes were detected at the injection site 13 weeks after subretinal delivery of AAV in a subset of treated animals. The injection site lesion size was quantified using **a.** fundus autofluorescence (FAF), **b.** fluorescein angiography (FA) and **c.** indocyanine green angiography (ICGA). In the Bonferroni-corrected subgroup analysis, stratified by AAV

dose level, there was generally no statistically significant difference between L1 and H2-treated eyes, except for ICGA in low dose treated eyes (3c.). Error bars: mean ±SD. *$P \leq 0.05$, ns: not significant **d.+e.** Peak clinical inflammatory activity after treatment was determined by quantifying the number of cells in the anterior chamber (ACC) and vitreous (VC) using the SUN (Standard of Uveitis Nomenclature) grading scheme. There was no difference in overt inflammation between L1 and H2-treated eyes. Error bars: median ± interquartile range. ns: not significant. Statistical significance was determined using the Wilcoxon rank sum test. L1: vector lot with low HCP (37ng/mL), H2: vector lot with high HCP (582ng/mL).
